# Association of Sleep Duration and Screen Time With Anxiety of Pregnant Women During the COVID-19 Pandemic

**DOI:** 10.3389/fpsyg.2021.646368

**Published:** 2021-04-20

**Authors:** Yuan Zhang, Yuge Zhang, Renli Deng, Min Chen, Rong Cao, Shijiu Chen, Kuntao Chen, Zhiheng Jin, Xue Bai, Jingyan Tian, Baofeng Zhou, Kunming Tian

**Affiliations:** ^1^Institute of Reproductive Health, Tongji Medical College, Huazhong University of Science and Technology, Wuhan, China; ^2^Department of Epidemiology, School of Public Health, Fudan University, Shanghai, China; ^3^The Fifth Affiliated Hospital, Zunyi Medical University, Zhuhai, China; ^4^Department of Obstetrics and Gynecology, Xiangyang No.1 People's Hospital, Hubei University of Medicine, Xiangyang, China; ^5^School of Basic Medicine, Tongji Medical College, Huazhong University of Science and Technology, Wuhan, China; ^6^Department of Health Education, Maternal and Child Hospital of Jinzhou, Jinzhou, China; ^7^Department of Gynecology and Obstetrics, Maternal and Child Hospital of Yanan, Yan'an, China; ^8^Department of Gynecology and Obstetrics, Maternal and Child Hospital of Bijie, Bijie, China; ^9^Department of Gynecology and Obstetrics, Maternal and Child Hospital of Shiyan Xiangyang, China; ^10^Department of Preventive Medicine, School of Public Health, Zunyi Medical University, Zunyi, China

**Keywords:** COVID-19, sleep, screen time, anxiety, pregnant women

## Abstract

The COVID-19 pandemic has dramatically changed the patterns of lifestyle and posed psychological stress on pregnant women. However, the association of sleep duration and screen time with anxiety among pregnant women under the backdrop of the COVID-19 pandemic scenario has been poorly addressed. We conducted one large-scale, multicenter cross-sectional study which recruited 1794 pregnant women across middle and west China. Self-reported demographic characteristics, lifestyle, and mental health status were collected from 6th February to 8th May 2020. We investigated the association of sleep duration and screen time with the risk of anxiety by multivariable logistic regression analysis and linear regression analysis after adjusting potential confounders. The dose-response relationship of sleep duration and screen time with anxiety was visualized using a cubic spline plot. Our data revealed that almost 35% of pregnant women suffered from anxiety during the COVID-19 pandemic. Sleep duration was dose-dependently associated with a lower risk of anxiety among pregnant women (OR = 0.41, 95% CI: 0.27–0.63), while screen time exhibited a conversed effect (OR = 2.01, 95% CI:1.00–4.39). Notably, sleep duration (≥8 h/day) synergistically combined with screen time (3–7 h/day) to diminish the risk of anxiety (OR = 0.70, 95% CI: 0.50–0.99). Taken together, sleep duration and screen time were independently and jointly associated with anxiety (*P* < 0.05). Therefore, promoting a more active lifestyle and maintaining higher sleep quality could improve the mental health of pregnant women, especially under public health emergency.

## Introduction

The novel coronavirus disease (COVID-19) has spread to 188 countries and more than 100,000,000 people have suffered from COVID-19^1^ Notably, the duration of the COVID-19 pandemic has the world on edge. Many countries adopted unprecedent strict quarantine and lockdown measures for effective control of COVID-19 (Zhang et al., 2020), which profoundly changed the pattern of lifestyle and contributed to sedentary behaviors. More importantly, the unpredictability and seriousness of this disease, together with social isolation, synergistically contributes to mental disturbance among the general population (Azim et al., 2020; Bao et al., 2020).

As a vulnerable population due to dramatic physiological and immunological changes during gestation (Narang et al., 2020), the mental health of pregnant women has increasingly drawn concern (Thapa et al., 2020). A recent study found that COVID-19 contributed to about 20.5% pregnant women suffering from anxiety symptoms in Wuhan (Liu et al., 2020). One reason for this is that pregnant women are seriously worried about being infected by COVID-19, which might impact the health of their fetus (Favre et al., 2020; Gross et al., 2020). More importantly, mental health problems are common in pregnant women (Yonkers et al., 2014). Notably, psychological stress during the pregnancy period could lead to various pregnancy complications, such as preeclampsia and low birth weight (Littleton et al., 2007; Qiao et al., 2012). Given the adverse outcomes of mental disorders, psychological stress surveillance, and early intervention of the anxiety of pregnant women under the COVID-19 pandemic is crucial.

Recently, a small sample cross-sectional study indicated that long-term lockdown and confinement at home elicited profound lifestyle changes, which are causing nearly half of pregnant women to suffer from anxiety (Corbett et al., 2020). Similarly, among Canadian pregnant women, COVID-19 markedly increased the prevalence of anxiety, especially for those with psychiatric history and low income (Berthelot et al., 2020). Furthermore, Wu et al. uncovered that pregnant women who are underweight, in employment, and have an economic burden were more likely to develop into anxiety in the Chinese population (Wu et al., 2020). Recently, data showed that sleep condition and sleep quality was associated with psychological stress (Lin et al., 2021). Importantly, a sedentary lifestyle also can contribute to various adverse health outcomes and is associated with poor sleep (Cao et al., 2011; The, 2019; Choi et al., 2020; Leger et al., 2020; van de Vegte et al., 2020). Nevertheless, the impact of this dramatic lifestyle alteration caused by the COVID-19 pandemic on psychological disorders has been poorly explored.

To fill this knowledge gap, we conducted a multi-center, large sample investigation that contains 1794 pregnant women from eight hospitals across four provinces in China. Our purpose was to investigate the prevalence of anxiety and further determine the independent and interactive association of sleep duration and screen time with anxiety among pregnant women. Our study could optimize psychological intervention strategies and empower the society to maximize the maintenance of the mental health of pregnant women by adopting healthy lifestyles.

## Methods

### Participants and Study Design

We designed the large-scale, multi-center, cross-sectional study which recruited pregnant women from eight hospitals of four geographically diverse areas (Hubei, Jiangxi, Shanxi, and Guizhou provinces) to determine the association of a COVID-19-mediated lifestyle with anxiety across middle and west China. Participants, who were outpatients, were recruited from 6th February, 2020 to 8th May, 2020. We initially recruited 1872 participants, and those among them who had infectious diseases (*n* = 2) and psychological diseases (*n* = 6) were excluded. We also excluded people with missing information about mental status, sleep duration, and screen time (*n* = 30), and people with an answer time of more than 40 min or <5 min (*n* = 40). After exclusion, a total of 1794 participants were finally enrolled in our study. The research was approved by the Ethics Committee of Tongji Medical College, Huazhong University of Science and Technology.

### Data Collection

#### Questionnaire

We developed a questionnaire that consists of five parts: socio-demographic information, sleep quality, screen time, physical activity, and anxiety assessment. Considering the government guidance to minimize the face-to-face contact during this COVID-19 pandemic, we collected related information from an online crowdsourcing platform named Wenjuanxing (www.wjx.com). The respondents were invited to scan the survey link using a smartphone and self-administrate to finish the questionnaire according to the instructions if the pregnant women confirmed their willingness to participate the survey. Informed consent was obtained from the participants before their participation in the study. Upon analysis, all questionnaires were subjected to quality audit.

#### Mental Health Assessment

Mental health was assessed using the 20 items Self-Rating Anxiety Scale. It composed of 20 items with four-point options (range: 1 = never, 2 = sometimes, 3 = often, 4 = very often) to capture the symptoms of anxiety. The SAS has been applied in Chinese populations in numerous studies, performing good validity and acceptable reliability (Kang et al., 2016; Ma et al., 2019; Wu et al., 2020). The Cronbach's α of SAS in the current study was 0.756. The anxiety score was calculated from the sum score of the 20 items. The cut-off point for anxiety was above 49, thus participants were categorized into healthy population (≤49) and anxiety population (≥50) based on anxiety score (Wu et al., 2020).

#### Assessment of Sleeping Duration and Screen-Time

Information regarding sleep duration and screen time was collected by a self-reported questionnaire. Sleep duration (h/day) was determined by asking the following question: “When did you usually go to sleep at night and wake up in the morning during COVID-19 pandemic?” Sleeping time was categorized into four groups: <6, 6–7, 7–8, ≥8 h. Screen-time was assessed by the question regarding “How many hours a day on average did you spend on mobile phone/computer/TV?” All screen-based activities were then summed for a total daily screen time (h/day) which was divided as: ≤2, 3–4, 5–6, 7–8, ≥ 8 h.

Commonly, 7–8 h is recognized as the better sleep duration for Chinese people. However, considering that the COVID-19 pandemic resulted in delayed and poor sleep patterns, and more time spent on social media (Deng et al., 2020), we set sleep duration for ≥8 h as the highest level. Sleep duration of <6 h was associated with a higher risk of adverse perinatal outcomes, therefore, <6 h was set as the lowest level for sleep duration (Cai et al., 2017). For screen time, previous research has found that screen time (>2 h) was associated with higher psychological stress (The, 2019). Therefore, we chose screen time (≤2 h) as the lowest level. Besides, given the calculated screen time in pregnant women during the COVID-19 pandemic, we divided the screen time of ≥8 as the highest group (Wu et al., 2020).

#### Covariates

Multiple covariates and potential confounders were controlled to measure the association of sleep duration and screen time with anxiety among pregnant women. These covariates included: pre-pregnant BMI, age, maternal type, occupation, gestational weeks, residence, psychologic situation during pandemic of COVID-19, timely prenatal examinations, cut off of health care products, household income during pandemic of COVID-19, rhythm of life during COVID-19, physical frequencies, screen time and sleep duration, education level, parity, pregnant complications, history of psychological diseases, and physical frequency. Given the fact that medical health workers and government employers have more responsibility for fighting COVID-19 in China and are more prone to have more higher risk to contact COVID-19 patients, thus they suffered more psychological stress than the general population. The occupation of whom was divided into government employee and non-government employee. The potential mediated effect of occupation on the association of interest was taken into consideration. Moreover, the severity of the COVID-19 pandemic determines the levels of isolation, quarantine, and proactive social distancing, which are closely related with lifestyle pattern and generate different mental pressure to the pregnant women. So, the residence in different provinces was categorized into three degrees during the COVID-19 pandemic according to the identified numbers of infected patients (low risk < 500; 500 < middle risk < 10,000; high risk > 10,000).

#### Statistical Analysis

All statistical analysis was performed by the SPSS (version 25.0, IBM, NY, USA) or R software (version 4.00, R core team). *P*-value was set at <0.05 as the threshold for statistical significance. In the descriptive analysis, the continuous and categorical variables were statistically described as Mean ± SD or n (%) in both the healthy population and anxiety group. The respondent characteristics in both the healthy group and anxiety group were analyzed through employing a *t*-test for continuous variables while a chi-square test was used for categorical variables.

Multivariable logistic regression analysis was used to estimate the crude and adjusted odds ratios (ORs) and 95% confidence intervals (CIs), and the dose-response effect was further visualized by the cubic spline. Moreover, the potential interactive effect of the association between sleep duration and screen time and anxiety through factorial analysis after controlling the covariates was performed.

Stratified analyses were conducted by baseline characteristics [including age (<25, ≥25 years), BMI (<25, ≥25 kg/m^2^), resident area (Hubei province, non-Hubei province), physical activity (≥2 times/week, <2 times/week), and education levels (less than undergraduate, undergraduate, and higher)].

The robustness of our results was assessed by sensitivity analyses, including (i) changing the confounder resident area as dichotomous variable (Hubei, non-Hubei); (ii) employing stratification analysis to control the potential confounders; (iii) conducting multiple linear regression analysis to further validate the relationship that sleep duration and screen time were closely associated with anxiety.

## Results

The characteristics of participants by anxiety status were showed in Table 1. The prevalence of anxiety among pregnant women during the COVID-19 pandemic was 34.5% (620/1794) in the total sample with a mean age of 27.44 ± 4.18. We found that the pregnant women with anxiety were more likely to have an interrupted nutrition additive intake due to strict quarantine and lockdown measures compared to mental health subjects. However, anxiety was associated with less exercise frequency, shortened sleep duration, and longer screen time. Additionally, there was no significant difference for pregnant women with or without anxiety regarding BMI, education, resident area, occupation, household income during COVID-19 pandemic, and prenatal examination.

**Table 1:** General characteristic of pregnant women in health and anxiety group.

**Variables**	**Health (*n* = 1174)**	**Anxiety (*n* = 620)**	***P*-value**
Age (years)	29.6 ± 4.2	29.0 ± 4.2	0.04
BMI (kg/m^2^)	21.4 ± 0.2	21.6 ± 0.3	0.11
Parity			0.23
Primiparae	688 (38.4%)	381 (21.2%)	
Multipara	486 (27.1%)	239 (13.3%)	
Education			0.29
High school and lower	691 (38.5%)	357 (19.9%)	
College and undergraduate	424 (23.6%)	221 (12.3%)	
Higher than undergraduate	60 (3.3%)	41 (2.4%)	
Residential areas			0.82
Low risk area	197 (11.0%)	106 (5.9%)	
Middle risk area	121 (6.7%)	58 (3.2%)	
High risk area	859 (47.8%)	456 (25.4%)	
Occupation			0.89
Government employee	314 (17.5%)	168 (9.3%)	
Non-government employee	860 (47.9%)	153 (8.5%)	
Household income during COVID-19 pandemic (CNY/month)			0.09
<5000	685 (38.2%)	398 (22.2%)	
5000–9999	346 (19.3%)	154 (8.6%)	
>10000	144 (8.0%)	67 (3.7%)	
Physical exercise (times/week)			<0.001
≤ 2	788 (43.9%)	475 (26.5%)	
>2	387 (21.5%)	144 (8.1%)	
Prenatal examination (times)			0.08
Normal	602 (33.6%)	290 (16.2%)	
Interrupted	573 (31.9%)	329 (18.3%)	0.004
1	270 (29.9%)	130 (14.4%)	
2	225 (24.9%)	132 (14.6%)	
3	43 (4.8%)	27 (3.0%)	
>3	35 (3.9%)	40 (4.5%)	
Healthy nutrition additive intake			<0.001
Normal	986 (54.9%)	466 (26.0%)	
Suspended	190 (10.6%)	152 (8.5%)	
Pregnancy complications			0.03
Yes	44 (2.5%)	37 (2.1%)	
No	1131 (63.0%)	582 (32.4%)	
Sleep duration (h/day)			<0.001
<6	62 (3.5%)	71 (4.0%)	
6–7	225 (12.6%)	120 (6.7%)	
7–8	550 (30.0%)	271 (15.7%)	
≥8	338 (18.8%)	157 (8.7%)	
Screen time (h/day)			<0.001
≤ 2	179 (10.0%)	54 (3.1%)	
3–4	321 (17.9%)	135 (7.5%)	
5–6	354 (19.7%)	194 (10.8%)	
7–8	168 (9.3%)	109 (6.1%)	
≥8	153 (8.5%)	127 (7.1%)	

**Figure 1:**
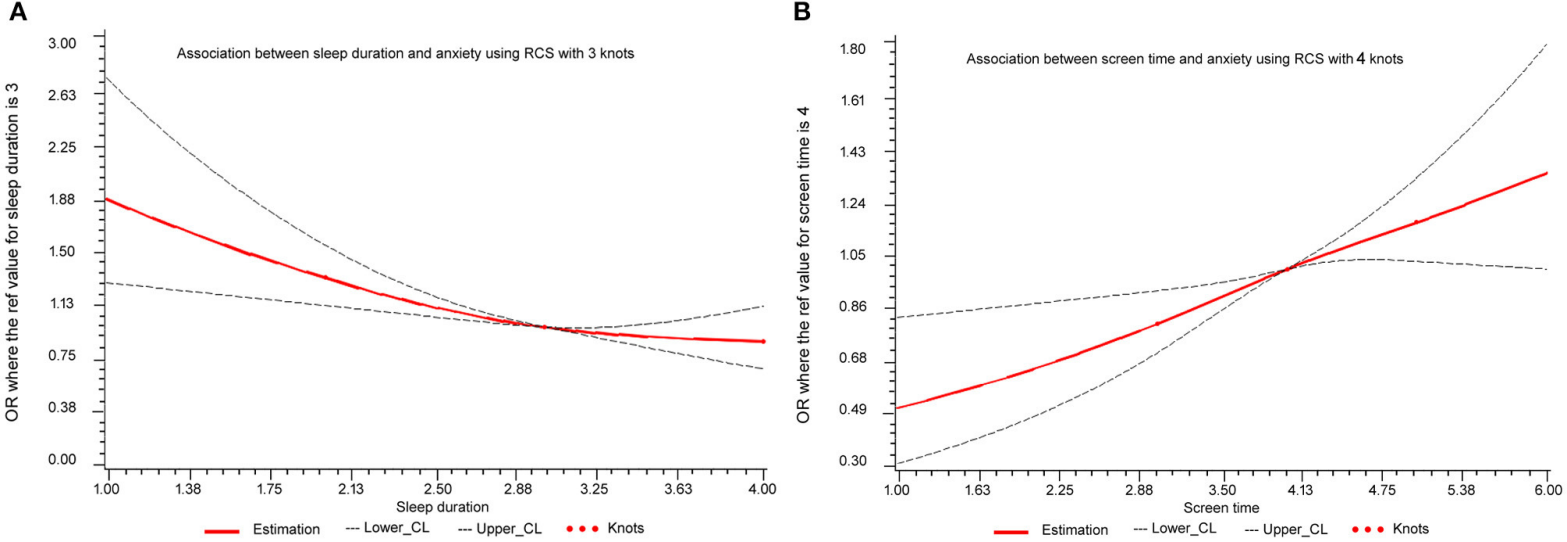
Dose-dependent association of **(A)** sleep duration and **(B)** screen time with anxiety.

Our data indicated that longer sleep duration was associated with lower anxiety while longer screen time was associated with higher anxiety. In the current study, the risk of anxiety exhibited a progressive decline trend with longer sleep duration (*P* trend <0.001) and sleep duration decreased an average of 50.2% compared to the reference group (<6 h/day) (OR = 0.41, 95% CI: 0.27–0.63) (Table 2). Conversely, screen time was significantly positively associated with anxiety (*P* trend <0.001) and the multivariate-adjusted OR (95% CI) >5 h/day was 2.01 (1.00–4.39), while the effect was less pronounced in people with screen time <5 h/day (Table 2). Furthermore, linear associations of sleep duration and screen time with anxiety were further presented by the spline curve in Figure 1. Cubic spline regression confirmed that the risk of anxiety might be higher with prolonged screen time and limited sleep duration. Notably, sleep duration (≥8 h/day) cooperated with screen time (3–7 h/day) and diminished the risk of anxiety (OR = 0.70 95% CI: 0.50–0.99) (Table 3).

**Table 2:** Odds ratios (95% CI) for anxiety according to sleep duration and screen time.

**Variables**	**Participants**	**Number of anxiety**	**Crude OR (95% CI)**	Adjusted OR (95% CI)^*^
Sleep duration				
<6 h	134	72	1.00	1.00
6–7 h	345	120	0.46 (0.30–0.69)^*^	0.41 (0.27–0.63)^*^
7–8 h	820	271	0.43 (0.29–0.62)^*^	0.37 (0.25–0.54)^*^
≥8 h	495	157	0.40 (0.27–0.59)^*^	0.35 (0.23–0.52)^*^
*P for trend*			<0.001	<0.001
Screen time				
≤ 2 h	234	55	1.00	1.00
3–4 h	456	135	1.37 (0.95–1.97)	1.38 (0.93–2.04)
5–6 h	547	194	1.79 (1.26–2.54)	1.76 (1.20–2.58)^*^
7–8 h	277	109	2.12 (1.44–3.12)^*^	1.98 (1.29–3.03)^*^
≥8 h	280	127	2.71 (1.85–3.98)^*^	2.22 (1.45–3.40)^*^
*P for trend*			<0.001	<0.001

*Adjusted for age, BMI, gestational weeks, residence, occupation, psychologic situation during pandemic of COVID-19, timely prenatal examinations, cut off of health care products, household income during pandemic of COVID-19, rhythm of life during COVID-19, education, physical frequency, screen time and sleep duration were adjusted for each other*.

* *These results were significant*.

**Table 3:** Odds ratios (95% CI) associated with the interaction of screen time and sleep duration on anxiety among pregnant women^*^.

**Screen time**	**Sleep duration <6 h**			**Sleep duration** **≥8 h**		
	**Number of anxiety (%)**	**Adjusted OR (95% CI)**	***P-value^**^#^**^***	**Number of anxiety (%)**	**Adjusted OR (95% CI)**	***P-value***
≥8 h	134 (41.61)	1.00		102 (43.59)	1.03 (0.71–1.48)	0.87
3–7 h	223 (32.65)	0.77 (0.57–1.03)	0.08	106 (33.12)	0.70 (0.50–0.99)	0.04^#^
≤ 2 h	34 (21.25)	0.45 (0.28–0.72)	0.001	21 (28)	0.57 (0.31–1.05)	0.07

* *Adjusted for age, BMI, gestational weeks, residence, occupation, psychologic situation during pandemic of COVID-19, prenatal examinations, cut off of health care products, household income during pandemic of COVID-19, rhythm of life during COVID-19, education, physical frequency*.

# *P-value is compared with reference (screen time ≥8 h and sleep duration <6 h)*.

The severity of COVID-19 in different provinces was hugely varied across China, among which Hubei province is the most severe one. Therefore, the robustness of the results was further validated after adjusted residence that was re-defined as a binary categorical variable (Hubei province and non-Hubei province) (Supplementary Table 1). Moreover, the results derived from the linear regression analysis also presented similar results (Supplementary Table 2).

Stratification analyses were subsequently conducted. There was a suggestion that the association between longer sleep duration and anxiety was stronger in individuals who were older than 25 years, without obesity, physically inactive, living in Hubei province, and those of higher education. However, no significant interactions were found with age, BMI, education, resident area, and physical activities (*P* for interaction > 0.05) (Table 4). The association between longer screen time and anxiety was stronger in individuals who were older than 25 years, without obesity, physically inactive, living in Hubei province, and those with higher education, however, no significant interactions were found with age, BMI, education, resident area, and physical activities (*P* for interaction > 0.05) (Table 5).

**Table 4:** Adjusted ORs (95% CI) for anxiety in people with sleep duration ≥8 vs. <6 h.

**Variables**	**OR (95% CI)**		***P for interaction***
Age		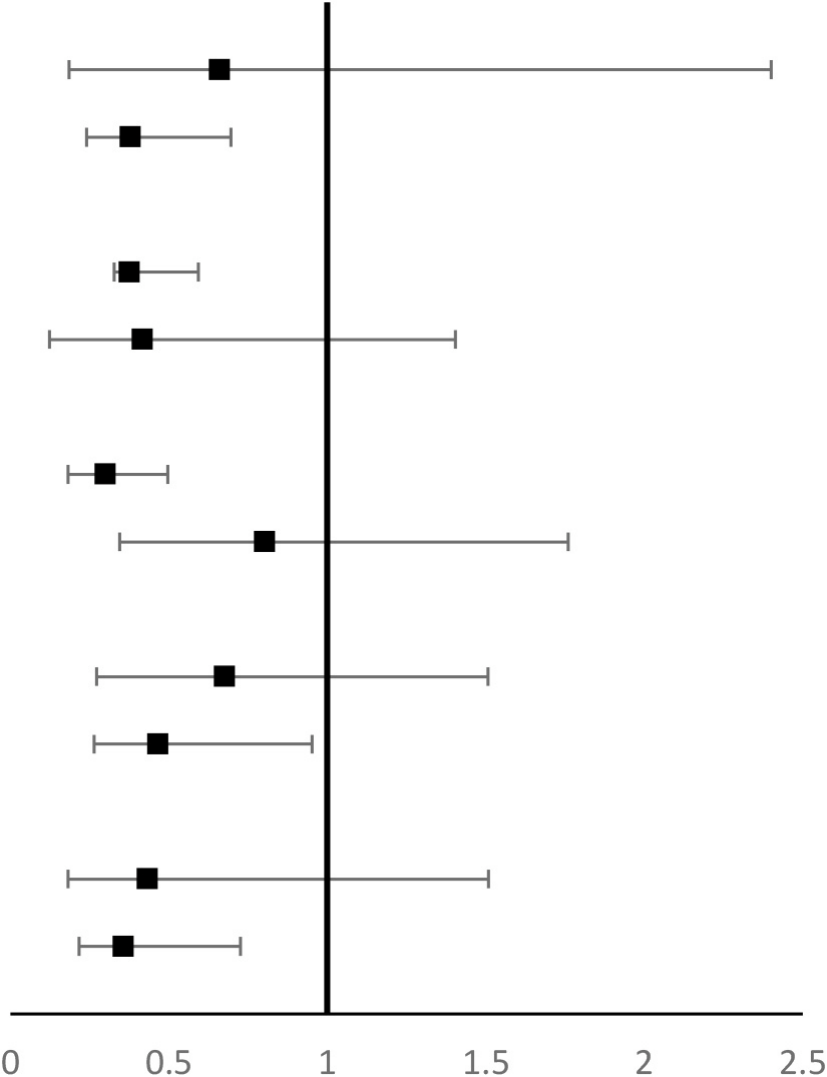	
≤ 25 years	0.66 (0.18–2.40)		0.35
>25 years	0.37 (0.24–0.69)		
BMI			
<25 kg/m^2^	0.37 (0.32–0.59)		0.57
≥25 kg/m^2^	0.41 (0.12–1.40)		
Resident area			
Hubei province	0.30 (0.18–0.49)		0.07
Non-Hubei province	0.80 (0.34–1.86)		
Education			
Less than undergraduate	0.67 (0.27–1.10)		0.82
Undergraduate and higher	0.44 (0.26–0.75)	
Physical activity			
≥2 times/week	0.43 (0.18-1.02)		0.92
<2 times/week	0.35 (0.21–0.58)		

*Adjusted odds ratios (95% CI) for anxiety in individuals with sleep duration (≥8 h) compared with the reference groups. All covariates were age, BMI, gestational weeks, residence, occupation, psychologic situation during pandemic of COVID-19, prenatal examinations, cut off of health care products, household income during pandemic of COVID-19, rhythm of life during COVID-19, education, physical frequency, and screen time. The reference groups were <6 h for sleep duration*.

**Table 5:** Adjusted ORs (95% CI) for anxiety in people with screen time ≥8 vs. ≤2 h.

**Variables**	**OR (95% CI)**		***P for interaction***
Age		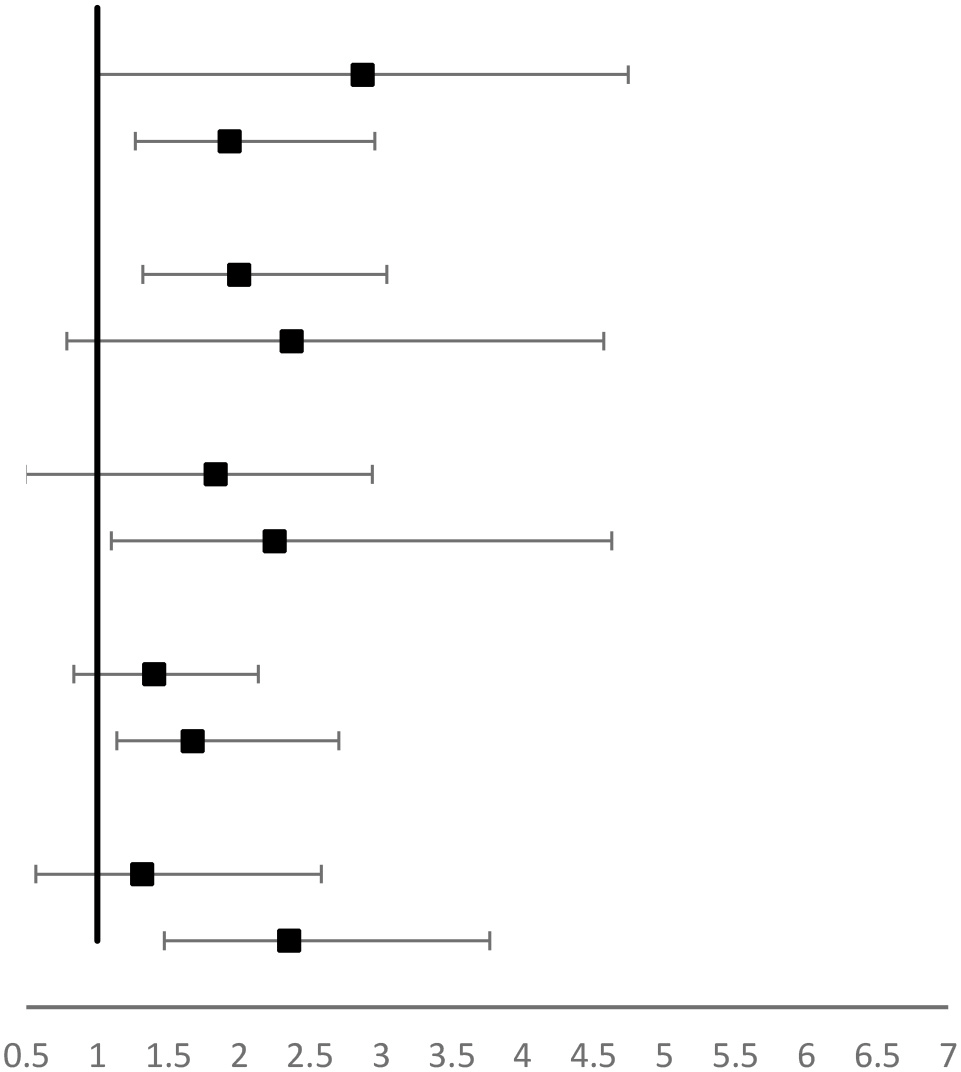	
≤ 25 years	2.87 (0.99–3.74)		0.39
>25 years	1.93 (1.26–2.95)		
BMI			0.26
<25 kg/m^2^	2.00 (1.32–3.04)		
≥25 kg/m^2^	2.37 (0.78–4.57)		
Resident area			
Hubei province	1.83 (0.49–2.93)		0.39
Non-Hubei province	2.25 (1.09–4.62)		
Education			
Less than undergraduate	1.40 (0.98–2.73)		0.95
Undergraduate and higher	1.67 (1.03-2.70)		
Physical activity			
≥2 times/week	1.32 (0.66–2.61)		0.74
<2 times/week	2.35 (1.47–3.76)		

*Adjusted odds ratios (95% CI) for anxiety in individuals with screen time (≥8 h) compared with the reference groups. All covariates were age, BMI, gestational weeks, residence, occupation, psychologic situation during pandemic of COVID-19, prenatal examinations, cut off of health care products, household income during pandemic of COVID-19, rhythm of life during COVID-19, education, physical activity frequency, and sleep duration. Each group adjusted for the other covariates except itself. The reference group was ≤ 2 h for screen time*.

## Discussion

The mental health of pregnant women has increasingly drawn concern. In this study, we observed that the prevalence of anxiety among pregnant women during the whole COVID-19 pandemic period in China was almost 35%. Furthermore, we found that a longer sleep duration (>6 h/day) was reversely associated with anxiety of pregnant women during the COVID-19 pandemic, while longer screen time (>5 h/day) was positively associated with anxiety. To our best knowledge, this is the first study to simultaneously explore the association of sleep duration and screen time with mental health under COVID-19 pandemic. These findings generated the implications for primary prevention of mental health problems by promoting healthy lifestyles among pregnant women.

We uncovered that the prevalence of anxiety among pregnant women during the COVID-19 pandemic in China was almost 35%, which was much higher than that of children (18.9%) (Xie et al., 2020) and health care workers in Wuhan (29.4%) (Kang et al., 2020), and of Singaporean (14.5%) (Tan et al., 2020). Regarding pregnant women in Canada who suffered from anxiety during the COVID-19 pandemic, the prevalence was 12.6%, which was also much lower compared with our study (Berthelot et al., 2020). This inconsistent data might be due to the fact that Berthelot et al. sub-divided psychiatric disorder into 13 subscale mental syndromes which might dilute the weight of anxiety in mental disorders, while our study focused on anxiety symptoms which might overestimate the weight/prevalence of anxiety. Moreover, Berthelot et al. recruited the participants ranging from 2nd April to 13th April 2020, a relatively short period which led to limited anxiety subjects, while we studied the anxiety status covering whole pandemic period in China. Conversely, Corbett et al. showed that ~50.7% of pregnant women were continuously worried about their health during the pandemic period. This study only recruited 71 participants who were second or third trimester, whereas the participants recruited in our study were covering the whole pregnancy (Corbett et al., 2020). Several studies showed the prevalence of anxiety in Wuhan and Chongqing city was 20.5 and 10.4%, respectively (Liu et al., 2020). Taken together, the above studies indicated that the COVID-19 pandemic might elicit psychological problems.

Against the unprecedented infectious disease pandemic in the past 100 years, the strict long-term lockdown at home and social distance were comprehensively/ubiquitously conducted. People underwent a rapid lifestyle transition from normal life to confined life, which elicited profound lifestyle changes (Corbett et al., 2020; Zhang et al., 2020) that made them spend more time on sleep and electronic products, which might cause potential influences on mental health during the COVID-19 pandemic. Wu et al. found that an exercise time of <7 h/week was associated with a 23% increase in depressive symptoms, which indicated that active lifestyle was closely related with mental health during the COVID-19 pandemic. However, no study has evaluated the association of sleep duration and screen time with anxiety among pregnant women under the COVID-19 pandemic scenario.

Cumulative data have indicated that both sleep quality and mental health are important for maintaining the health of pregnant women (Qiao et al., 2012; Loy et al., 2020). Sleep quality closely impacts the mental health of pregnant women (van der Zwan et al., 2017; Difrancesco et al., 2019), and sleep duration and anxiety interactively contribute to pregnancy complications (Tomfohr-Madsen et al., 2019). Thus, the association of sleep duration with anxiety is warranted to further investigate under the public health emergency. Both cross-sectional and longitudinal studies have indicated that a sleep duration of <8 h/day and bad sleep quality can increase anxiety during pregnancy (Yu et al., 2017). However, previous evidence regarding the association of sleep duration with mental disorders was assessed under normal life. COVID-19 markedly raised mental disorder risk and changed the average person's sleep pattern. A meta-analysis indicated that 34% population suffered from sleep disturbances (Deng et al., 2020). Importantly, COVID-19 induces a later and longer sleep pattern on weekdays, lower levels of social jetlag, and a delayed chronotype (Leone et al., 2020). Though the sleep condition and quality were associated with the mental health of pregnant women in Shenzhen (Lin et al., 2021), the participants in this study were restricted to Shenzhen, only from one city. To date, little is known regarding the association between sleep duration and mental health in a representative population. Our work conducted a large population and multi-central study to address this important concern, and we found that longer sleep duration might be dose-dependently associated with lower anxiety in pregnant women during the COVID-19 pandemic under our research scenario.

Screen viewing is increasingly prevalent and has become a part of common sedentary behavior globally, which might cause mental disorders (The, 2019). Importantly, the association of a sedentary lifestyle and COVID-19-induced psychological stress among pregnant women has not yet been explored. Our study revealed that longer screen time was associated with increased risk of anxiety among pregnant women during the COVID-19 pandemic. The plausible explanation might be that longer screen time makes pregnant women exposed to more dubious information about rapidly increased numbers of infection rate and mortality, which resulted in fear and concern among them (Wang et al., 2019). Currently, the association of screen time with mental health mainly focuses on adolescents. In Chinese adolescents, a screen time of >2 h/day was significantly associated with anxiety (Cao et al., 2011; Wu et al., 2016). Importantly, this effect was more apparent when combined with insufficient physical activity (Cao et al., 2011). The notion of independent and jointed effects of screen time and physical activity with anxiety was further validated in Canadian adolescents (Herman et al., 2015). It is noteworthy that the above studies mainly focused on the combined effects of screen time and physical activity on mental disorders, while the interactive effect of screen time and sleep duration has yet not been addressed. Notably, sleep duration (>7 h/day) cooperated with screen time (3–6 h/day) to diminish the risk of anxiety. Our data could provide scientific evidence for guiding pregnant women toward being more active and less sedentary/screen-based in their daily life, especially under the public health emergency.

## Strengths and Limitations

We conducted a large-scale and multi-center study that recruited heterogeneous 1794 participants from eight hospitals across middle and western China, which covered the high risk, middle risk, and low risk areas of COVID-19. The first study that investigated the mental health of pregnant women in China during COVID-19 pandemic failed to recruit people from Wuhan, the severest place of COVID-19 in China. By contrast, 1,050 subjects of our study were enrolled from Wuhan. Thus, our data could realistically reflect the mental health and changed lifestyle of participants during COVID-19 pandemic, which is an important strength of our study. Furthermore, we not only determined the independent association of sleep duration and screen time with anxiety, but also investigated the interactive association of sleep duration and screen time with anxiety among pregnant women. However, some limitations still should be disclosed. First, due to strict confinement and quarantine measures, we failed to collect information face-to-face, and all demographic data and mental health situations were self-reported, thus the report bias cannot be avoided. Second, the present study was a cross-sectional design, which made us fail to determine casual relationships of screen time and sleep duration with anxiety. Third, the screen time derived from our study was total screen viewing time, so we failed to determine the association between device-specific screen viewing time and anxiety. Moreover, the association of sleep quality with anxiety among pregnant women was not explored.

## Conclusion

In summary, longer sleep duration was associated with lower anxiety among pregnant women while screen time was positively associated with anxiety among pregnant women during the COVID-19 pandemic. However, further longitudinal research on the causal relationships between sleep duration and screen time with mental health are needed to verify the results.

## Data Availability Statement

The data presented in this article is available on request. Requests to access these datasets should be directed to Kunming Tian, nonstandstill@163.com.

## Ethics Statement

The studies involving human participants were reviewed and approved by Ethics Committee of Tongji Medical College, Huazhong University of Science and Technology. Written informed consent for participation was not required for this study in accordance with the national legislation and the institutional requirements.

## Author Contributions

KT and YugZ conceived the study. YuaZ and YugZ searched the data. KT and YugZ analyzed the data. YuaZ and KT gave advice on meta-analysis methodology and wrote the draft of the paper. RC, MC, ZJ, XB, JT, and BZ contributed to collecting data, or revising the paper. KT is the guarantors of this work and take responsibility for the integrity of the data and the accuracy of the data analysis. RD, SC, and KC carefully revised the paper. All authors read and approved the final manuscript.

## Conflict of Interest

The authors declare that the research was conducted in the absence of any commercial or financial relationships that could be construed as a potential conflict of interest.

## Footnotes

^1^WHO coronavirus Disease (COVID-19) Dashboard: https://covid19.who.int/.
