# Supplementary material for: Association of Sleep Duration and Screen Time With Anxiety of Pregnant Women During the COVID-19 Pandemic
Source: Front Psychol. 2021 Apr 20;12:646368. doi: 10.3389/fpsyg.2021.646368 (PMC8093759; doi:10.3389/fpsyg.2021.646368)
Supplement: Supplementary file 1 [file Data_Sheet_1.docx]

**Supplementary materials:**

Table 1 Odds ratios(95%CI) for anxiety according to sleep duration and screen time*

| Variables | Participants | Number of anxiety | Crude OR (95%CI) | Adjusted OR (95%CI)^*^ |
| --- | --- | --- | --- | --- |
| sleep duration |  |  |  |  |
| <6 hours | 134 | 72 | 1.00 | 1.00 |
| 6-7 hours | 345 | 120 | 0.47(0.31-0.71) | 0.48(0.31-0.75) |
| 7-8 hours | 820 | 271 | 0.43(0.29-0.63) | 0.47(0.32-0.71) |
| ≥8 hours | 495 | 157 | 0.39(0.26-0.58) | 0.38(0.25-0.58) |
| *P for trend* |  |  | <0.001 | <0.001 |
| Screen time |  |  |  |  |
| ≤2 hour | 234 | 55 | 1.00 | 1.00 |
| 3-4 hours | 456 | 135 | 1.36(0.76-3.34) | 1.47(0.70-3.51) |
| 5-6 hours | 547 | 194 | 2.07(0.99-4.32) | 2.01(0.90-4.45) |
| 7-8 hours | 277 | 109 | 2.39(1.13-5.08) | 2.26(0.99-5.13) |
| ≥8 hours | 280 | 127 | 3.01(1.42-6.37) | 2.53(1.11-5.74) |
| *P for trend* |  |  | <0.001 | <0.001 |

* We changed the confounder residence as dichotomous variable (Hubei, non-Hubei) and used logistic regression to further confirm whether the relationship between sleep duration and screen time and the risk of anxiety was robust. Adjusted for age, BMI(continuous), gestational weeks, residence, occupation, psychologic situation during pandemic of COVID-19, prenatal examinations, cut off of health care products, household income during pandemic of COVID-19, rhythm of life during COVID-19, education, physical frequency, screen time and sleep duration were adjusted for each other.

.

Table 2 Relationships between sleep duration, screen time and anxiety scores*

|  | Scores | |
| --- | --- | --- |
| Variables | β (95% CI) | *P* |
| sleep duration | -0.40(-0.62-0.17) | <0.001 |
| screen time | 0.26(0.10-0.42) | <0.001 |

*We used multivariate linear regression to explore the relationship between sleep duration and screen time and the risk of anxiety. Adjusted for age, BMI, gestational weeks, residence, occupation, psychologic situation during pandemic of COVID-19, prenatal examinations, cut off of health care products, household income during pandemic of COVID-19, rhythm of life during COVID-19, education, physical frequency, screen time and sleep duration were adjusted for each other.
